# Parting ways: *Pan-Homo* divergence revisited

**DOI:** 10.1007/s10329-026-01269-w

**Published:** 2026-06-18

**Authors:** João d’Oliveira Coelho, Robert L. Anemone, René Bobe, Susana Carvalho

**Affiliations:** 1 Science Department, Gorongosa National Park, Chitengo, Sofala Mozambique; 2 Primate Adaptations, Landscapes and Evolutionary Origins (PALEO), CIBIO-Universidade do Porto, Campus de Vairão, Vila do Conde, Portugal; 3 Centre for Functional Ecology (CFE), Universidade de Coimbra, Coimbra, Portugal; 4 Department of Anthropology, University of North Carolina at Greensboro, Greensboro, NC USA; 5 Interdisciplinary Centre for Archaeology and Evolution of Human Behaviour (ICArEHB), Universidade do Algarve, Faro, Portugal

**Keywords:** Hominin origins, *Pan* split, Divergence dates, Primate fossil record, Late Miocene, Metascience

## Abstract

The timing of divergence between hominins and the bonobo-chimpanzee clade has been at the core of palaeoanthropological debate for over a century. The earliest molecular studies indicated divergence times ranging from 5 Ma to as recently as 1.3 Ma. This study critically reviews the trends of time estimates published between 1967 and 2023, and analyses how these are supported or rejected by the current molecular and fossil records. We compiled 202 divergence estimates and defined three distinct thresholds based on fossil evidence at 4.4 Ma (*Australopithecus anamensis* and *Ardipithecus ramidus*), 6.2 Ma (*Orrorin tugenensis* and *Ardipithecus kadabba*), and 7.2 Ma (*Sahelanthropus tchadensis*). We then used these thresholds to filter out molecular estimates that are too young to fit the fossil record. Overall, the data suggests a divergence event within the late Miocene, with each threshold pushing it further back, 8.63–6.38, 10.33–7.81, and 10.95–8.81 Ma, respectively. We use a quadratic regression to demonstrate that estimates have been slowly shifting from ~ 6 Ma to ~ 8.5 Ma over the past 56 years. A Bayesian meta-analysis of genomic estimates filtered by our most consensual threshold (i.e., assuming *Australopithecus* belongs to Hominini) indicates that the split must have occurred early in the late Miocene, most likely before 7 Ma (~ 99.5% posterior probability) with a pooled effect of 8.69–7.28 Ma. We conclude that, despite an initial bias towards younger estimates, the molecular timing for the last common ancestor (LCA) of *Pan*-*Homo* has been progressively approaching the intervals suggested by the current fossil record.

**Supplementary Information:**

The online version contains supplementary material available at https://doi.org/10.1007/s10329-026-01269-w.

## Introduction

Huxley (1863) and Darwin (1871) proposed that humans and African apes form a clade on the basis of morphological and biogeographic evidence. This hypothesis gained wide support after it was empirically demonstrated by the immunochemistry studies of Goodman in the 1960s (1961, 1963). In the same decade, Sarich and Wilson (1967) used immunological data to estimate that the divergence between humans and the African apes (a group now recognized as paraphyletic, comprising the lineages of *Pan* and *Gorilla*) had occurred at around 5 Ma by assuming a calibration point of 30 Ma for the split between Hominoidea and Cercopithecoidea. This was based on the assumption that some genetic changes arose at nearly constant rates, and there “may thus exist a molecular evolutionary clock” (Zuckerkandl and Pauling 1965, p. 148). Later, by defining a split calibration of 75 Ma between Perissodactyla and Primates, the divergence of humans and other apes was readjusted by Wilson and Sarich (1969) to between 5 and 4 Ma. In the following decades, some molecular biologists have estimated even more recent times for this evolutionary event, such as 1.3 Ma (Goodman 1981), or 2.8–0.3 Ma (Ruvolo 1997), which at the time challenged the hominin status attributed not just to *Australopithecus* but also to *Paranthropus* as well as multiple species of early *Homo*.

The hypothesis of a very recent divergence event between humans and African apes sharply contrasts with the much older dates proposed by palaeontologists prior to the advent of molecular techniques. For example, Keith (1920, p. 507) argued that humans diverged from other apes before the Miocene, which at the time was generally placed near ~ 27 Ma (Berggren et al. 1985), rather than its current, well-constrained, base at 23.04 Ma (Walsh 2008). Consistent with this view, Simpson (1959) proposed a divergence event at ~ 32 Ma. Later, and with access to more fossil evidence, Pilbeam (1967) suggested that the hominin clade could have originated at any time between 35 and 20 Ma. However, by the early 1970s, when the phylogenetic issues of the *Gorilla*–*Pan*–*Homo* trichotomy were yet to be fully resolved, Vincent Sarich asserted that the field of molecular biology had decisively won the debate: “[t]o put it as bluntly as possible, I now feel that the body of molecular evidence on the *Homo*-*Pan* relationship is sufficiently extensive so that one no longer has the option of considering a fossil specimen older than about eight million years a hominid no matter what it looks like” (1971, p. 76).

Indeed, in the 1970s–1990s it was not uncommon to cast doubt on the fossil evidence due to molecular models, e.g.: “this dating [2.68 Ma] may pose a problem for the widely believed hypothesis that the bipedal creature *Australopithecus afarensis* […] was ancestral to man and evolved after the human-ape splitting.” (Hasegawa et al. 1985, p. 160). With the acceptance of molecular clock techniques, the scenario of a human–ape divergence happening around the early Miocene, defended by palaeontologists up to the 1960s, rapidly lost supporters. It is worth noting, however, that such scenario developed when the fossil evidence available was substantially more limited than the record available today. Likewise, the earliest molecular studies were also carried with access to far less data quantity and quality than available now and with far less sophisticated techniques. Thus, many palaeontologists remained sceptical of early molecular studies suggesting the divergence must have happened during the Pliocene. Nevertheless, consilience between molecular clocks and the fossil record after decades of disagreement can happen, as demonstrated by the case of the origin of placental mammals (Goswami 2012). As it tends to happen with conflicting views stemming from different methodological paradigms (Wilson 1998), some expected that the evidence of both disciplines would eventually coalesce at some intermediate time, in the middle to late Miocene (Senut 2010, 2015).

The main problem introduced by the Pliocene and the 6–4 Ma molecular divergence estimates is that they are in direct conflict with most early hominin fossil discoveries of the past three decades. There are now several likely candidates for early hominin status spanning from 5.4 Ma to possibly 7.4 Ma, such as *Ardipithecus kadabba* (Haile-Selassie 2001), *Orrorin tugenensis* (Senut and Pickford 2001) and *Sahelanthropus tchadensis* (Brunet et al. 2002). Additional Miocene findings, in this case showing strong affinities with the Panina subtribe, include the 6.1 Ma “Cheboit ape” molar (Senut 2010) and, possibly, the BAR 91’99 lower molar from the 12.5 Ma Ngorora Formation (Pickford and Senut 2005). The latter would be very difficult to fit with current divergence estimates if it is indeed a member of the Panina. There are also late Miocene fossils from Africa that are likely to belong to the gorilline stem lineage, e.g., the 8 Ma remains of *Chororapithecus abyssinicus* (Suwa et al. 2007), a 6–5 Ma canine from Nkondo, Uganda (Pickford et al. 1988), and the 5.9–5.8 Ma dental finds attributed to the Kapsomin large ape (Senut 2010, 2015). *Nakalipithecus nakayamai*, dated to 9.9–9.8 Ma (Kunimatsu et al. 2007), has also recently been proposed to be a member of the tribe Gorillini (Katoh et al. 2016). Other late Miocene African apes that have not been ascribed to either Hominina, Panina or Gorillini include a second, ~9.85 Ma hominid (indet.) from Nakali (Kunimatsu et al. 2016), as well as *Samburupithecus kiptalami* from the 9.5 Ma Samburu Hills of Kenya (Ishida and Pickford 1997). It should be noted, however, that the primate fossil record is, overall, relatively scarce, with only 7% of fossil record preservation at the species level (Tavaré et al. 2002). Furthermore, this record is particularly thin in the late Miocene of Africa (d’Oliveira Coelho et al. 2021).

Molecular studies for the Panina-Hominina (hereby *Pan*/*Homo*) divergence over the past six decades have produced widely varying estimates, from as recent as 1.3 Ma (Goodman 1981) to as early as 14.0 Ma (Romero-Herrera et al. 1973). However, there has been no formal meta-analysis examining how these estimates have changed over time or how well they are supported by the current fossil record and novel molecular techniques (Jensen-Seaman and Hooper‐Boyd 2008; but see Bradley 2008). We address this gap by integrating the state of the art of both molecular and fossil data. Historically, molecular estimates sometimes diverged from the fossil record, highlighting discrepancies between the two sources of evidence (Raaum et al. 2005). Rather than treating one type of data as superior to the other, a more productive approach is to assess multiple scenarios that combine both molecular and palaeontological evidence.

Following the principle that if a given species or well-dated fossil X is part of the hominin (or panin) clade, any divergence time estimate that is more recent and thus excludes X is inconsistent with that species’ placement. Palaeontological thresholds therefore act as minimum hard bounds supported by specific fossil evidence and should be incorporated into meta-analytic approaches (see *Fossil thresholds* section in “Materials and methods”).

Allowing multiple scenarios is particularly important given the lack of taxonomic consensus for some of the earliest proposed hominins. Fossil-based thresholds serve to constrain molecular estimates, preventing implausibly recent values from dominating meta-analyses or regression models. For example, using the first threshold at 4.4 Ma (supported by *Australopithecus anamensis* and/or *Ardipithecus ramidus*) (Bobe and Wood 2022) ensures that molecular clock models inferring a *Pan*/*Homo* divergence more recent than 4.4 Ma are treated as incompatible with the fossil evidence. The same logic applies to the second threshold (*Orrorin tugenensis* and/or *Ardipithecus kadabba*) and the third threshold (*Sahelanthropus tchadensis*), using the respective dating and associated taxa. By incorporating palaeontological constraints in this way, we can assess multiple scenarios while preventing clearly unsupported estimates from biasing the results.

Crucially, the use of palaeontological thresholds also raises interpretive limitations that must be made explicit. Estimating the true temporal range of any fossil species is inherently challenging, as it is extremely unlikely that the first appearance datum (FAD) corresponds exactly to the speciation event. Moreover, the first detectable skeletal apomorphies within a lineage can take several million years to evolve, meaning that the earliest known fossils often postdate the actual divergence (Marshall 2008, 2017, 2019). Against this backdrop, our study aims to: (1) Quantify the historical trajectory of *Pan*/*Homo* divergence estimates through a comprehensive meta-analysis of six decades of molecular data; (2) Evaluate the fossil-molecular lag by testing how quickly landmark palaeontological discoveries are integrated into molecular clock calibrations; and (3) Determine the most parsimonious divergence interval by synthesizing phylogenomic data with conservative fossil thresholds to resolve current disciplinary discrepancies.

## Materials and methods

### Pre-processing

To understand what the consensus in molecular biology is regarding the time of divergence for the *Pan*–*Homo* pair, we created a new database documenting the published estimates of this splitting event. As a starting point, we downloaded a dataset containing molecular time estimates available at http://timetree.org (TimeTree 5) (Kumar et al. 2017, 2022; Craig et al. 2024). This dataset contains five variables (Time, Title, Reference, Year, Data) and 81 entries for the *Pan*/*Homo* pair published between 1985 and 2020. We reorganized and increased the number of variables, ending up with the following features for data analysis: Reference, Title, Year, Month, Max, Estimate, Min, Material, ErrorMetric, and Notes. The first four variables are identifiers, and the Max-Estimate-Min triplet represents the corresponding range of values estimating the *Pan*/*Homo* split. Material is a variable that identifies what was analysed (e.g., mtDNA, genome, etc.). ErrorMetric is a binary Bayes/SE variable to record if the interval of estimates was calculated with a 95% Credible Interval (Bayesian), or a Standard Error (frequentist). SE also includes studies that only presented a 95% Confidence Interval (CI). During database imputation, frequentist CI was transformed into SE by the following operation: * SE=(upperCI-lowerCI)/3.92*, in order to achieve standardization of frequentist errors. In a normal distribution, a 95% interval encompasses approximately ± 1.96 standard errors from the mean. Therefore, the total width of the interval is twice that, i.e., 3.92. Bayesian 95% credible intervals (BCI) were kept in the database without transformations. Finally, the Notes column contains general observations and remarks about the articles.

After scanning the studies collected at timetree.org to confirm the data and extract new variables, we also corrected several entries containing mistakes (e.g., mismatches in estimate values) in the original dataset (documented in Notes), and removed one entry from the timetree.org set that was considered an outlier—a paper focused on Archaean lifeforms (David and Alm 2011) that contains an estimate of the *Pan*/*Homo* divergence at 139.7 Ma. To expand our dataset, we added new entries cited in these same papers and acquired further novel entries by searching Google Scholar using combinations of “divergence”, “homo”, “pan”, “hominin”, “molecular”, and “split”. When papers had multiple models, we recorded them as multiple entries (1 per model/output), unless models had extremely similar outputs and could straightforwardly be grouped and averaged by similar sets of techniques. To enhance reproducibility, our final database is publicly available at GitHub and can be used and updated by others interested in maintaining the project or working on future iterations of it (https://github.com/Delvis/Pan-Homo). Currently, it has a total of 12 variables and 202 entries, each corresponding to an individual molecular time estimate for the *Pan*/*Homo* divergence published between 1967 and 2023.

### Fossil thresholds

For the data analysis, we defined three different palaeontological thresholds based on the fossil and stratigraphic evidence. The first is a 4.4 Ma threshold supported primarily by *Australopithecus anamensis* (estimated origination = 4.37 Ma) (Du et al. 2020), a widely accepted hominin. But notice that *Ardipithecus ramidus* was also found at 4.493–4.3 Ma deposits (Simpson et al. 2019) giving further support to a 4.4 Ma minimum bound. The second is a 6.2 Ma threshold based on *Orrorin tugenensis* first appearance datum (FAD = 6.1 Ma) (Roche et al. 2013) and *Ardipithecus kadabba* (FAD = 6.3 Ma) (Simpson et al. 2015). The third is a 7.2 Ma threshold for *Sahelanthropus tchadensis*, known from 7.34 to 7.10 Ma deposits (Lebatard et al. 2010) that have been suggested to possibly reach 7.4 Ma (McDougall and Feibel 2003). We rely on each species FAD, since they can be used as an absolute minimum estimate for the divergence of two distinct taxa (Marshall 2008). Yet, given that all FADs underestimate the divergence times they are estimating (Marshall 1990), all the palaeontological thresholds we provide might be too conservative.

The 4.4 Ma threshold: By the mid-1990s *Australopithecus anamensis* (Leakey et al. 1995) and *Ardipithecus ramidus* (White et al. 1994) had been described as new species of hominins older than 4 Ma. More precisely, the FAD of *Au. anamensis* is ~ 4.2 Ma (Ward et al. 2020) while *Ar. ramidus* is known from 4.51 to 4.32 Ma (White et al. 2009). However, recent work has suggested that the origination date of *Au. anamensis* may be 4.37 Ma (Du et al. 2020), or even reach 4.6 Ma (Bobe and Wood 2022), and new fossil discoveries push the FAD of *Ar. ramidus* to 4.8 Ma (Simpson et al. 2019). Recent phylogenetic studies further confirm the consensus regarding the classification of both species as hominins (Mongle et al. 2019). Moreover, the Lothagam mandible (KNM-LT 329) from 4.9 to 4.4 Ma is likely to belong to *Australopithecus* (Kissel and Hawks 2015). There is also the Tabarin mandible (KNM-TH 13150) and more material recently uncovered from 5 to 4.5 Ma deposits in Baringo, that have been attributed to a new early Pliocene hominin taxon, described as *Orrorin praegans* (Pickford et al. 2022).

The 6.2 Ma threshold: In 2001, discoveries in Ethiopia and Kenya of late Miocene hominins described as *Ardipithecus kadabba* (Haile-Selassie 2001) and *Orrorin tugenensis* (Senut and Pickford 2001) challenged many traditionally held views about human evolution, such as the savanna-based models of hominin origins (Crompton et al. 2010). If we accept either of these species as hominin, with FADs around 5.8 (Haile-Selassie et al. 2004) and 6.1 Ma (Roche et al. 2013; Senut et al. 2018), respectively, we must accept that the *Pan/Homo* divergence happened some time before 6 Ma. *Orrorin tugenensis* femur shows some of the earliest evidence for facultative bipedalism, and shares traits with later *Australopithecus* (Almécija et al. 2013). Regarding *Ardipithecus kadabba*, while the majority of material is very well-constrained to 5.77–5.54 Ma (WoldeGabriel et al. 2001), it should be noted that now there is also 6.3 Ma material from Gona, Ethiopia, attributed to cf. *Ardipithecus kadabba* (Simpson et al. 2015). Concurrently, there is also the Cheboit ape, possibly belonging to the *Pan* lineage at 6.1 Ma (Senut 2010). Consequently, this threshold was defined at 6.2 Ma.

The 7.2 Ma threshold: The discovery of *Sahelanthropus tchadensis* (Brunet et al. 2002), found between two strata dated to 7.34 ± 0.12 and 7.10 ± 0.14 Ma (Lebatard et al. 2010), challenged recent split estimates. Despite its controversial status as a hominin (Wolpoff et al. 2002, 2006; Pickford 2005; Macchiarelli et al. 2020; Meyer et al. 2023), *Sahelanthropus*’ known morphology seems to share some derived features with hominins such as the reduction of the canine that may be correlated with a non-honing C-P3 complex, and a more anteriorly positioned *foramen magnum*, likely related to an incipient form of bipedal locomotion (Zollikofer et al. 2005). Moreover, two recently described antimeric ulnae and one left femur share more morphological traits with *Ardipithecus*, *Orrorin*, *Australopithecus*, *Paranthropus* and *Homo* than with extant and Miocene apes (Daver et al. 2022). Therefore, we regard *S. tchadensis* as the oldest record of a plausible hominin. Notice that even if *S. tchadensis* belonged to the Panina subtribe rather than Hominina (Meyer et al. 2023), the logic to support this fossil threshold would still apply. This is because any fossil from either lineage would contribute to establishing the minimum age boundary of their split. Regardless of its taxonomic status, recent analyses confirm that *Sahelanthropus* exhibits key bipedal adaptations including strong femoral antetorsion, a hominin-like femoral tubercle for iliofemoral ligament attachment, and a derived gluteal complex, while retaining *Pan*-like limb proportions and climbing adaptations, supporting its status as an early habitual biped (Williams et al. 2026).

If we consider that Cenozoic mammal species have an average duration of 2.3–1.6 Ma (Marshall 2017), together with the patchiness of the early Pliocene and late Miocene of Africa (virtually no hominin evidence older than 7.3, or at 7.1–6.3 and 5.2–4.8 Ma), we can further argue that the values chosen for thresholds are conservative. This is mostly due to the unlikelihood of the sampled FAD matching the true origination date in species known from few localities and individuals (Marshall 2019; Du et al. 2020; Bobe and Wood 2022). This is further complicated by the fact that the period ranging from 9 to 7.2 Ma in Africa is represented by a single geological formation (Chorora Fm, Ethiopia) and locality with only ca. 200 vertebrate fossils (Katoh et al. 2016). In a recent study, Bobe and Wood (2022) used confidence intervals to calculate the origin of taxa, and estimated *Sahelanthropus* speciation at 9 Ma while *Orrorin* and *Ardipithecus* origins were placed at 8 Ma. Under that scenario the fossil thresholds defined above can be seen as practical but conservative minimum age bounds, because they are ultimately intended to filter out estimates that are incompatible with hypotheses assigning specific extinct taxa to the hominin lineage.

### Regression analyses

After curating the new database of split dates, we performed several regression analyses using the divergence estimates as the outcome variable, with year-and-month of publication as its covariate. In this study, publication date can be seen as a general proxy variable for technological, statistical, and theoretical advancements on phylogenetical inference and divergence estimation models. These include the transition from constant to relaxed molecular clocks, the exponential increase in genomic data quality, and the implementation of more sophisticated Bayesian models that better account for life-history variables like generation time. This allows us to understand if any specific trends exist that support the common intervals cited in human evolution textbooks that usually consider the *Pan*/*Homo* split to have occurred at 6–4 or at 7–5 Ma (e.g., Boyd and Silk 2009; Larsen 2009; Foley and Lewin 2013). To better grasp the nuances of the dataset, five different regression models were built: Ω) full-dataset model; (A) miscellaneous; (B) mitochondrial; (C) nuclear DNA; (D) genome. The first regression analysis includes all data points available. Reg-A combines the phenetic estimates (immunological and DNA-DNA hybridization) with RNA estimates. Reg-B is focused on mitochondrial derived studies, while Reg-C is based on sequences of nuclear DNA. The last regression model only includes studies of divergence using genomic data—these were only possible after the genome of *Pan troglodytes* had become publicly available in 2005 (Gunter and Dhand 2005; Waterson et al. 2005). Approaching the dataset from different perspectives is crucial in order to have an unbiased estimate of the divergence event between the Hominina and Panina subtribes.

### Meta-analyses

Additionally, we interpreted the dataset and the subsets filtered by the different thresholds using a Bayesian multilevel regression for meta-analysis as implemented in the brms package (version 2.16.3) for R version 4.5.2 (Bürkner 2017, 2018; R Core Team 2025). We are not aware of other meta-analyses of molecular divergence studies in the literature (Supplementary Information, S2).

The different divergence time estimates in the literature have been built using different assumptions. Consequently, they have different types of clock models, material sampled (DNA, mtDNA, etc.), datasets used and partitioning schemes, generation times, mutation rates, min-max bounds, priors used, among other variables. The point could be made that they cannot be directly comparable. However, following Cumming’s definition of an effect size as anything we might be interested in measuring across studies, including “a mean, a difference between means, a percentage, a median, or a correlation […] standardized value, such as Cohen’s * d* […] or a regression coefficient, path coefficient, odds ratio, or percentage of variance explained” (Cumming 2013, p. 38) we *assume* that any molecular divergence estimate obtained from the literature can be used as a proxy to calculate effect size, since, despite being obtained through different methods, they are all estimating the same phenomenon (in this case, divergence between *Pan* and *Homo*) in the same units of measurement (Ma).

Furthermore, our primary meta-analysis exclusively incorporates genome-derived divergence estimates from 2005 onwards (i.e., same as Reg-D). Contrary to previous studies, these estimates share several methodological similarities and exhibit a more cohesive and comparable initial set of assumptions. In our meta-analysis both studies with frequentist standard errors (SE) or Bayesian Credible Intervals (BCI) were included. Assuming a Gaussian shape of the posterior distribution, the formula * SE=(upperBCI-lowerBCI)/3.92* can be applied to convert Bayesian 95% Credible Intervals into SE, similarly to what is usually done to transform frequentist confidence intervals into standard errors (but in the frequentist scenario, the assumption here enounced is not required).

### Data and materials availability

The raw data HomoPanDivergences.csv contains published divergence estimates of *Pan*/*Homo* for replicating the data analyses presented in this manuscript in a fully reproducible manner. It is available for download at https://github.com/Delvis/Pan-Homo as a legacy version. The code used for generating all analyses, figures, and tables is also publicly available at the same GitHub repository.

## Results

### Dataset structure

**Fig. 1:**
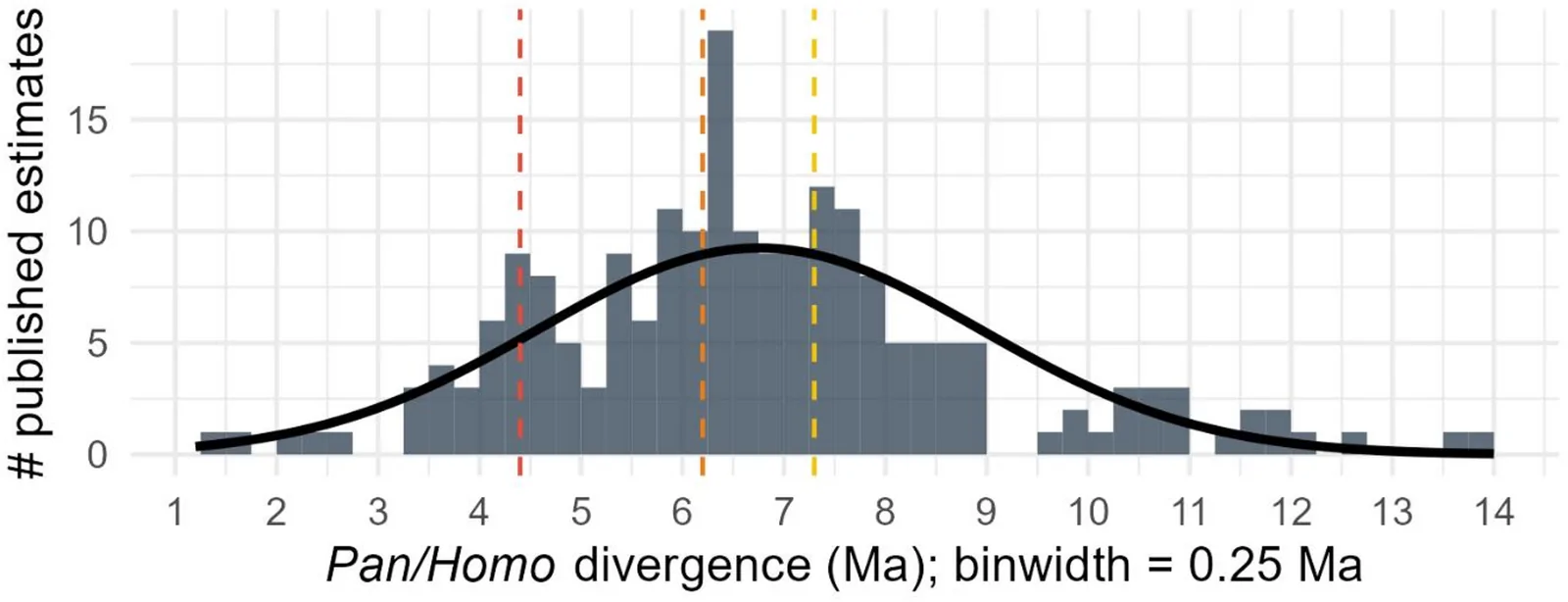
Molecular estimates histogram. Dashed vertical lines represent the *fossil thresholds*. Note that instead of following a normal distribution, studies seem to cluster in excess around important fossil discoveries, and there is an unexpected gap of mean-estimates at 9–9.5 Ma

The dataset contains 202 molecular divergence estimates for the *Pan*/*Homo* pair, ranging from 1.3 to 14 Ma, with a mean estimate of 6.76 ± 0.15 Ma and a standard deviation of 2.2 Ma (Table 1). With this standard deviation allowing for a divergence as recent as 4.59 Ma, all the early putative hominins (*Ardipithecus*, *Orrorin*, *Sahelanthropus*, and even arguably the earliest *Australopithecus*) appear to be incompatible with the central tendencies observed in the dataset. Moreover, the data is positively skewed (skewness = 0.66) which indicates that most values are concentrated on the left side of the distribution (i.e., skew towards younger dates), as well as slightly leptokurtic, entailing that there are more estimates than expected around the mean. While this pattern could potentially reflect a clustering of estimates around the true biological date of divergence, it can also suggest a systematic reporting bias due to calibration toward fossil-informed minimums. Overall, the molecular estimates tend to cluster at points in time coinciding with the palaeontological thresholds we defined. These thresholds match FAD dates established for the major early hominin fossil discoveries, and form three statistically anomalous peaks in the histogram likely due to underestimation combined with calibration issues shifting the estimates to the fossil-informed minimum (Fig. 1).

**Table 1:** Descriptive statistics of the *Pan*/*Homo* divergence estimates

Filtered by threshold											
Subset	n	Mean	sd	Median	Mad	Min	Max	Range	Skew	Kurtosis	se
Full	202	6.76	2.18	6.52	1.77	1.3	14	12.7	0.61	0.84	0.15
4.4 Ma	177	7.21	1.91	6.9	1.48	4.5	14	9.5	1.12	1.26	0.14
6.2 Ma	120	8.06	1.72	7.56	1.28	6.29	14	7.71	1.38	1.34	0.16
7.2 Ma	73	8.95	1.68	8.3	1.05	7.3	14	6.7	1.16	0.4	0.2
*Subsets for regression analyses*											
Reg-A	24	6.41	2.92	5.88	2.38	1.3	14	12.7	0.75	0.3	0.6
Reg-B	62	6.62	2.26	6.46	1.3	2.5	13.7	11.2	0.88	0.9	0.29
Reg-C	65	6.22	1.65	6.29	1.47	1.55	11.75	10.2	0.3	1.25	0.21
Reg-D	51	7.76	1.98	7.74	1.28	3.69	12.71	9.02	0.21	−0.01	0.28

Includes the distributions for the full dataset, and for the three subsets using hominin fossil thresholds used in meta-analyses, as well as the distributions of the subsets used in regression

### The 4.4 Ma threshold: the last *Ardipithecus* and the first *Australopithecus*

Using the most consensual of the palaeontological thresholds at 4.4 Ma, we discovered that a total of 25 divergence estimates in the dataset of *Pan*/*Homo* split dates fall outside of this bound (Table 1). Notably, 14 of these were published after the fossil material of *Ardipithecus ramidus* and *Australopithecus anamensis* was described in 1994–95 (White et al. 1994; Leakey et al. 1995). This strongly suggests that there is sometimes a noticeable lag between genetic divergence studies and the incorporation of new palaeontological findings into their model assumptions. Some of these divergence estimates are younger than 2.8 Ma and could even be rejected based solely on the fossil record of *Paranthropus* or early *Homo*, long recognized as hominins. Excluding molecular models that do not meet the 4.4 Ma threshold would result in an interval of 8.63–6.37 Ma for the *Pan*/*Homo* divergence. Note how strikingly this deviates from the more commonly cited interval of 6–4 Ma popularized in introductory text books of human evolution and biological anthropology (e.g., Hoffecker 2017, p. 126), and in the first molecular studies (e.g., Wilson and Sarich 1969).

### The 6.2 Ma threshold: *Orrorin tugenensis* and *Ardipithecus kadabba*

The average minimum age for the *Pan*/*Homo* split is at 5.62 Ma for our full dataset of molecular divergences (Table 1; Supporting Information Fig. S1), a date too recent to be supported by the fossil evidence assuming that either the *Orrorin* or the *Ardipithecus* lineages represent early hominins. After applying a 6.2 Ma threshold to the estimates in order to reduce potential bias towards young values, we obtain instead an interval of 10.30–7.77 Ma for the hominin split. This result is very close to the interval of 10–7 Ma that White et al. (2009) proposed based solely on palaeontological and stratigraphic evidence from the African late Miocene. Nevertheless, this threshold is likely not strict enough as the majority of published estimates cluster just before it. In fact, there are over twice as many estimates at 6.25–6.5 Ma as would be expected by a normal distribution curve (Fig. 1). While this pattern could potentially reflect a clustering of estimates around the true biological date of divergence, it more likely reflects the influence of earlier age estimates for key late Miocene fossils and commonly applied minimum-age constraints near 6 Ma in molecular clock models (e.g., Raaum et al. 2005; Israfil et al. 2011; Das et al. 2014; Yaxley and Foley 2019), which tend to generate divergence estimates that cluster just above these calibration boundaries.

### The 7.2 Ma threshold: *Sahelanthropus tchadensis*

**Fig. 2:**
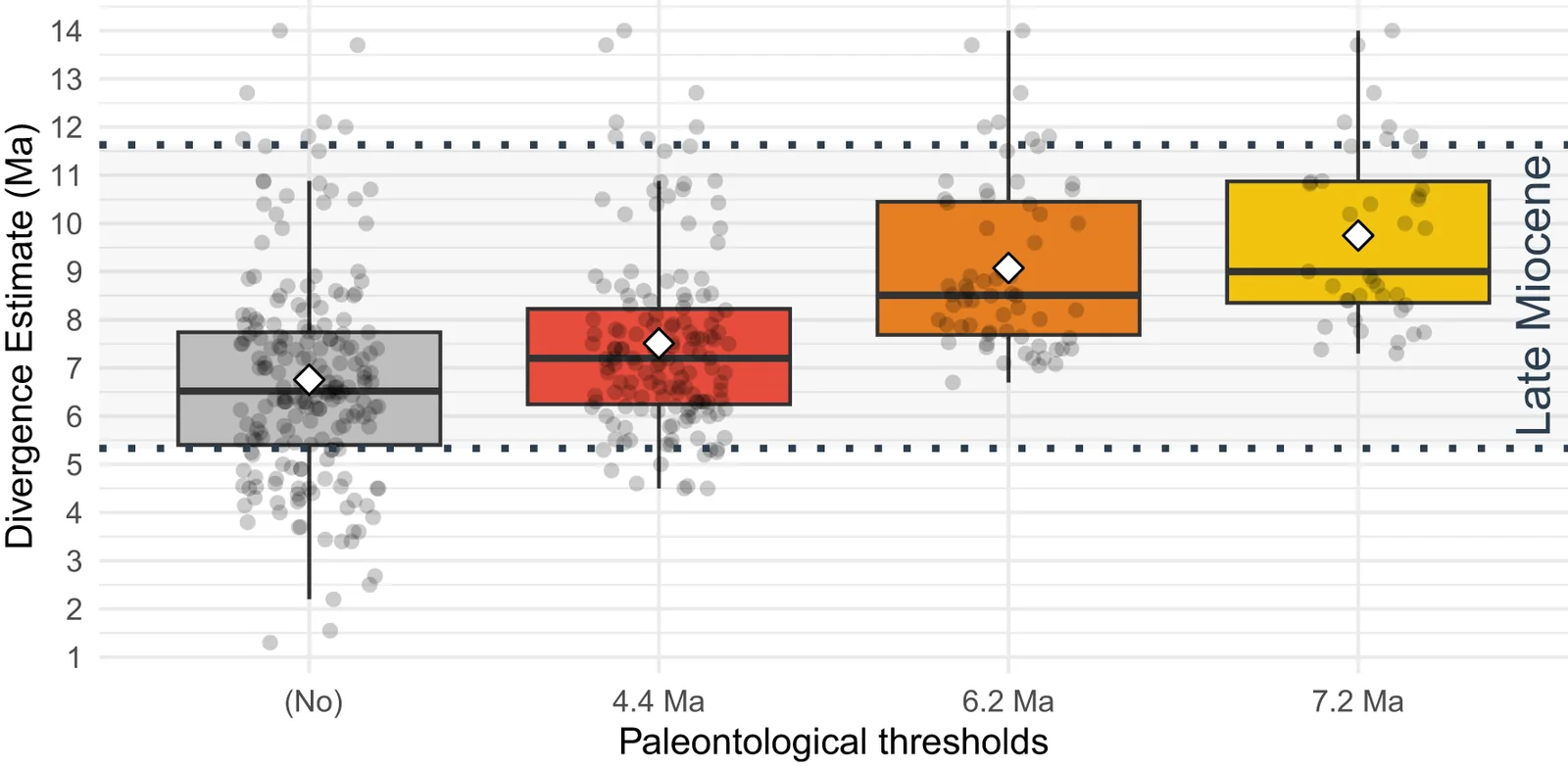
Interquartile range boxplots for divergence estimates filtered by different fossil thresholds. The arithmetic means and the medians are represented by white diamonds and black bars, respectively. All boxplots fit within the late Miocene (11.6–5.3 Ma) sub-epoch, represented by a light-grey band between horizontal dotted-lines

When we set 7.2 Ma as an absolute minimum for the divergence of hominins, we find that 130 out of the 202 molecular clock estimations pose challenges. If we filter by the available minimum estimates instead (i.e., lower estimate of the confidence interval), 86.11% of the observed divergence estimates for *Pan*/*Homo* in the literature are incompatible with this threshold and thus with *Sahelanthropus*. The exclusion of these estimates would increase the arithmetic mean estimate of *Pan*/*Homo* divergence models from 6.79 (7.91–5.62) Ma to 9.88 (10.95–8.81) Ma, aligning more closely with the current palaeontological data (see Marshall 2019). Figure 2 provides boxplots summarizing the results of all threshold analyses. The arithmetic means consistently indicating older temporal ranges than the respective medians is a behaviour typical of log-normal distributions (Supplementary Information S1) and can thus be interpreted as evidence of a skew towards recent divergences (Fig. 2).

### Regression analyses of molecular estimates from 1967 to 2023

**Fig. 3:**
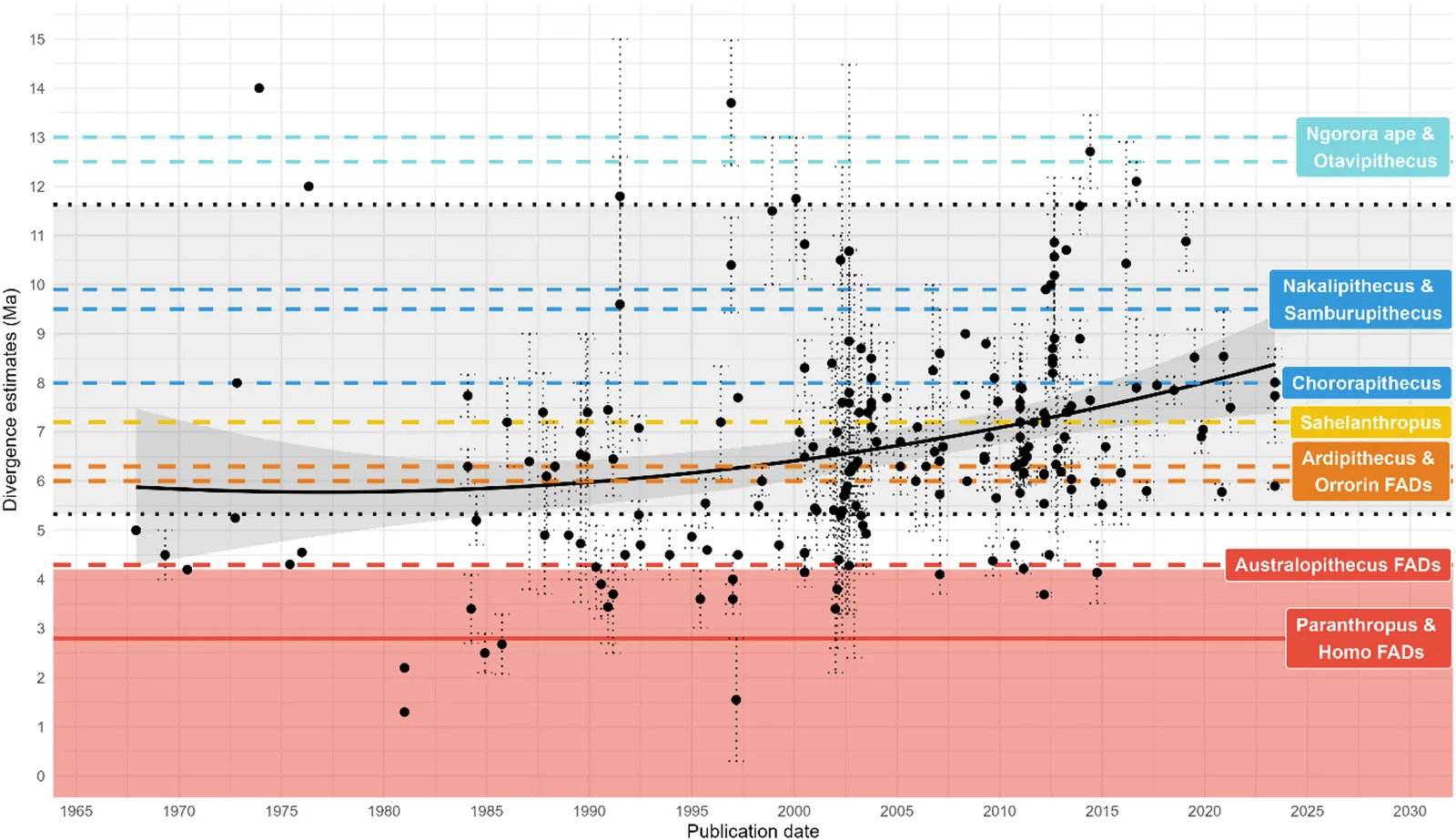
Trend of the “full-dataset model” fitting the sample of Panina/Hominina split estimates by date of publication. The late Miocene period is shadowed and within the horizontal dotted black bars. Dotted error-bars are standard errors, presented when available (studies with confidence or credible intervals were transformed to SE)

Regression models with 95% confidence intervals show patterns within the divergence estimates over a 56-year period (Fig. 3; Table 2Ω). In the “full-dataset model” polynomial regression we have an early stage with much uncertainty associated (1960–1980 s) due to the large disparity of values and scarcity of studies. For this period, the regression curve suggests a divergence date around 6 Ma. After 1985, the lower limit of the confidence interval reaches and remains within the boundaries of the late Miocene. Following the discovery of the earliest hominins at the turn of the century (Senut and Pickford 2001; Haile-Selassie 2001; Brunet et al. 2002), plus the first phylogenomic studies after the chimpanzee genome was published (Gunter and Dhand 2005; Waterson et al. 2005), and the renewed interest in the hominoid slowdown hypothesis (Steiper et al. 2004; Elango et al. 2006), the trend towards older divergence estimates accelerated significantly, and as of 2023, the regression suggests a split between *Pan* and *Homo* at 8.4 (9.4–7.4) Ma.

**Table 2:** Summary statistics for the regression analyses

(Ω) Divergence estimates of the full dataset			
Predictors	β	CI	p
(Intercept)	6.76	6.47 to 7.05	**< 0.001**
Date [1st degree]	8.36	4.23 to 12.49	**< 0.001**
Date [2nd degree]	2.98	−1.15 to 7.11	0.157
Observations	202		
R^2^/R^2^ adjusted	0.083/0.073		

(A) Divergence estimates				(B) Divergence estimates			
Predictors	β	CI	p	Predictors	β	CI	p
(Intercept)	−82.1	−283.29 to 119.02	0.406	(Intercept)	−70.89	−202.1 to 60.3	0.284
Date	0.04	−0.06 to 0.15	0.371	Date	0.04	−0.03 to 0.10	0.242
Observations	24			Observations	62		
R^2^ / R^2^ adjusted	0.037/−0.007			R^2^/R^2^ adjusted	0.023/0.006		

(C) Divergence estimates				(D) Divergence estimates			
Predictors	β	CI	p	Predictors	β	CI	p
(Intercept)	−94.48	−217.8 to 28.8	0.131	(Intercept)	−173.00	−427.81 to 81.8	0.179
Date	0.05	−0.01 to 0.11	0.108	Date	0.09	−0.04 to 0.222	0.160
Observations	65			Observations	51		
R^2^ / R^2^ adjusted	0.041/0.025			R^2^/R^2^ adjusted	0.040/0.020		

(Ω) full-dataset model; (A) miscellaneous model; (B) mitochondrial model; (C) nuclear DNA model; and (D) phylogenomic model

Bold values indicate statistically significant results (*p* < 0.05)

**Fig. 4:**
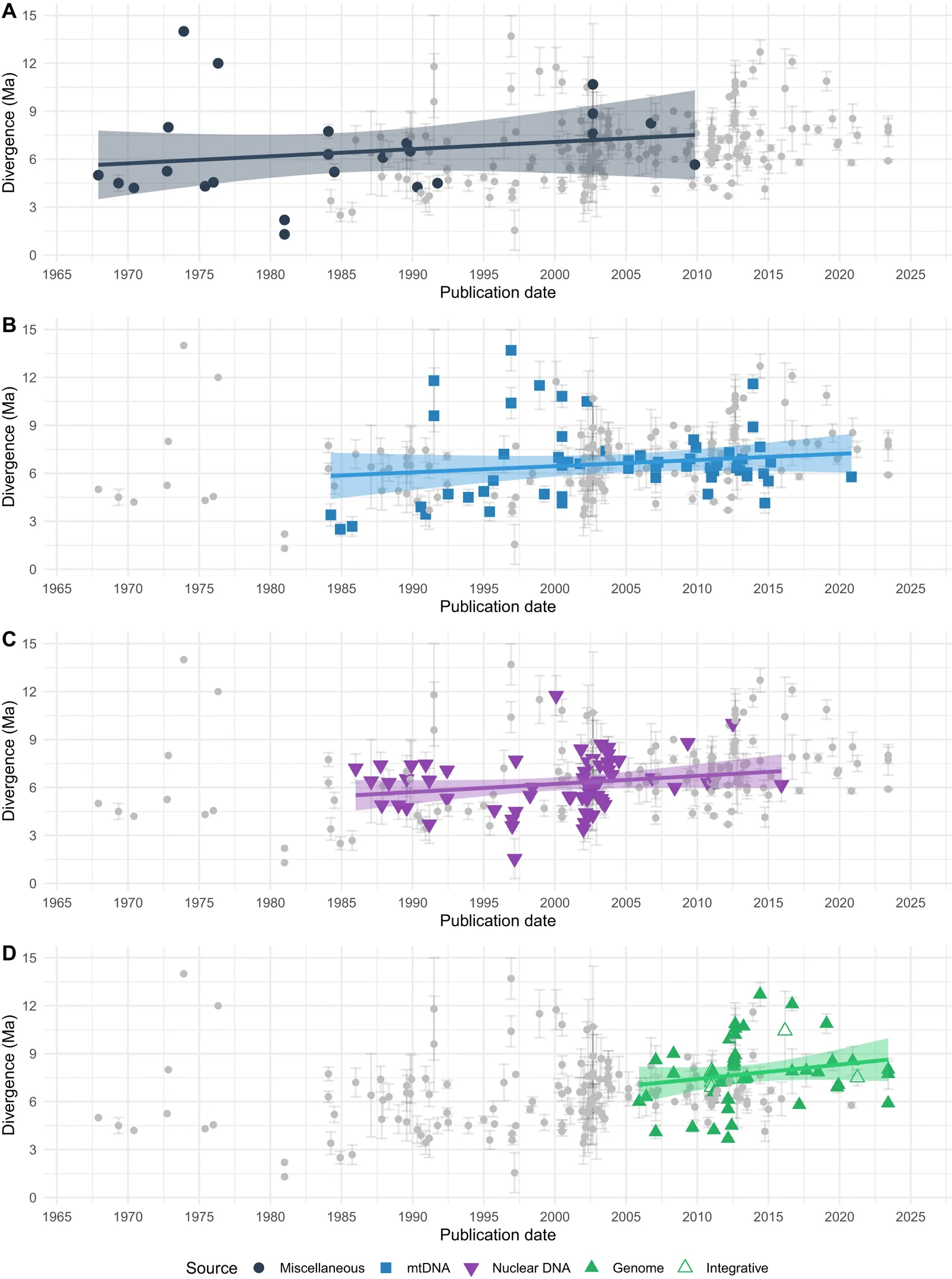
Linear regressions for different types of source datasets. **A** Includes early phenetic studies (immunological, DNA-DNA hybridization, etc.), but also later studies with virogenes and RNA; **B** Mitochondrial datasets from early studies with selected sequences to later complete mitochondrial genome studies; **C** Split estimates based on nuclear DNA sequences; and **D** Large phylogenomic studies (also includes ‘total evidence dating’, i.e., integrative models)

Considering the different types of genetic material used across studies (nuclear vs. mitochondrial), or the resolution of the datasets (selected sequences vs. genome-wide analysis), and the fact that the early immunological studies are phenetic instead of phylogenetic (i.e., they cannot distinguish between derived and plesiomorphic similarity), we provide results to different sets of regressions focusing on different source material (Fig. 4). While all sub-regressions Reg-A, Reg-B, Reg-C and Reg-D showed positive slopes, with divergence estimates suggesting older intervals of the late Miocene as date of publication progresses, none seems to exhibit a significant relation between date of publication and estimate. Summary statistics for each model are available on Table 2A–D.

The first sub-model is based on the early immunological and DNA-DNA hybridization techniques of the 1960–1980 s, as well as some later RNA-based studies (Fig. 4A). It has the smallest signal-to-noise ratio of the four sub-models, including a negative adjusted R^2^, and the smallest *n* = 24 (Table 2A). The second and third models based on mitochondrial (Fig. 4B; Table 2B) and nuclear DNA (Fig. 4C; Table 2C) divergence studies have similar outputs, showing gentle positive slopes towards older geological dates for the split.

Finally, the “genomics-specific model” uses a linear regression to analyse the estimation trend of every genomic data point in the database (Fig. 4D). It has the highest positive slope of all regression analyses, increasing quickly from 7.1 (8.2–5.9) Ma since the publication of the chimpanzee genome (Gunter and Dhand 2005) towards 8.6 (9.9–7.3) Ma as of 2023. The regression formula obtained for genomic-only datasets indicates divergence estimates published between 2005 and 2023 have been getting older on average by 90 Ka per year (Table 2D).

### Bayesian meta-analyses

To combine evidence from multiple studies, we performed a meta-analysis of the literature under the framework that a meta-analysis is a special case of Bayesian multilevel regression modeling (Supplementary Information S2). Using the 4.4 Ma, the 6.2 Ma, and the 7.2 Ma thresholds we obtained meta-estimates ranging from 7.23 to 6.58 Ma (Fig. S2, Table S1), 8.03–7.35 Ma (Fig. S3, Table S2), and 9.12–8.17 Ma (Fig. S4, Table S3), respectively. Notice that even the last would be supported by some of the most recently published divergence studies e.g., 8.52 (9.81–7.58) Ma (dos Reis and Yang 2019).

**Fig. 5:**
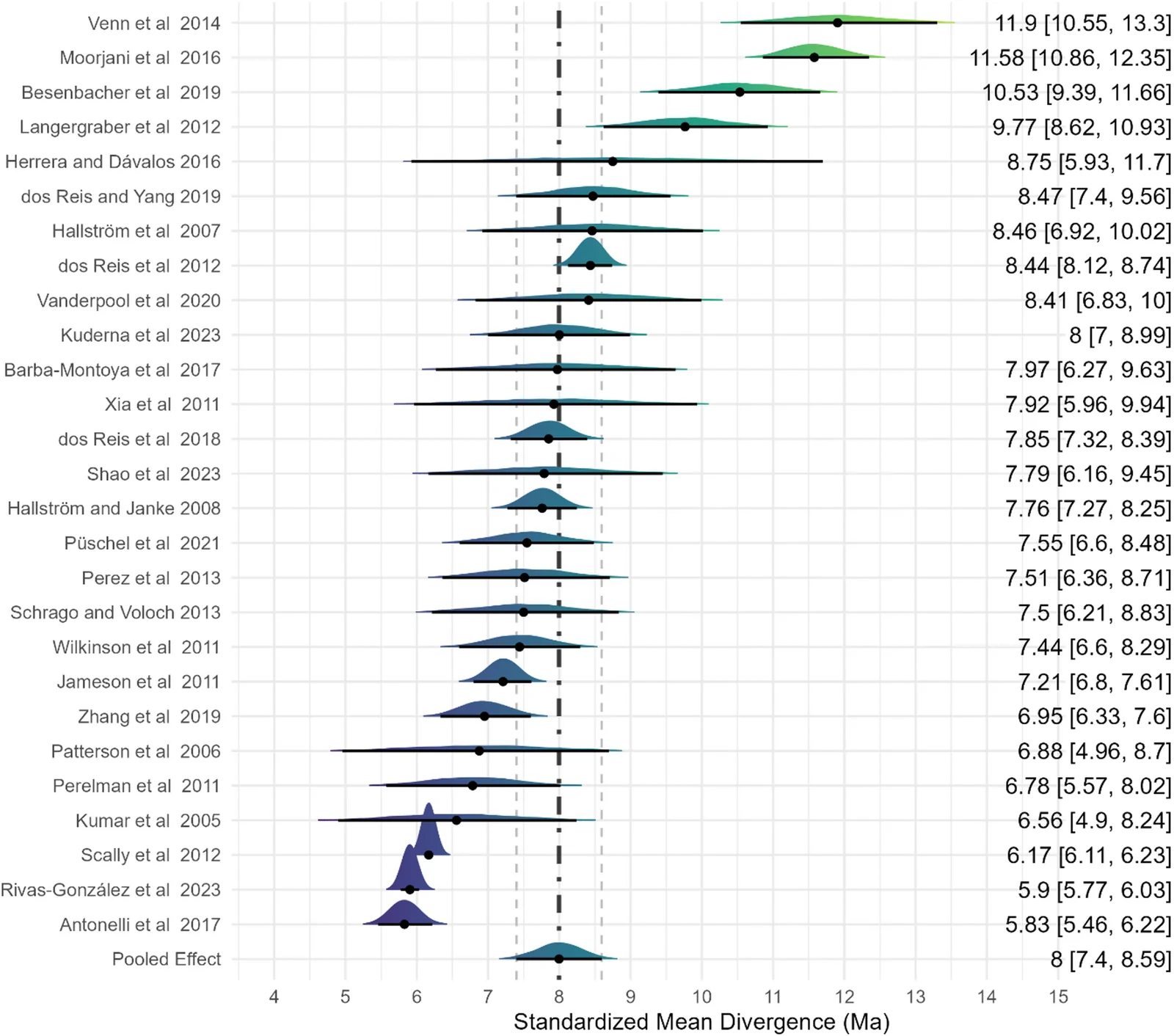
Meta-analysis of the Pan/Homo phylogenomic divergence estimates. Forest plot depicting the specific effect size and sampled posterior distribution of genome-based studies

After sub-setting the dataset to only include phylogenomic studies (i.e., same as in Reg-D) and also older than 4.4 Ma (the most consensual and conservative threshold) in order to exclude studies that reject both *Australopithecus anamensis* and *Ardipithecus ramidus* status as hominins, we obtained a meta-estimate of 8.0 Ma with a distribution of the pooled effect ranging from 8.69 to 7.28 Ma (Fig. 5; Table 3).

**Table 3:** Summary statistics for the Bayesian meta-analysis for the phylogenomic studies

7.2 Ma threshold	Divergence | SE ~ 1 + (1 | Study)	
Predictors	Estimates	CI (95%)
Intercept Std. dev.	8.0 1.70	7.28–8.69 1.25–2.35
Random effects		
τ_00 Study_	0.71	
ICC	0.79	
N_Study_	27	
Observations	41	
Conditional R^2^	0.750	

**Fig. 6:**
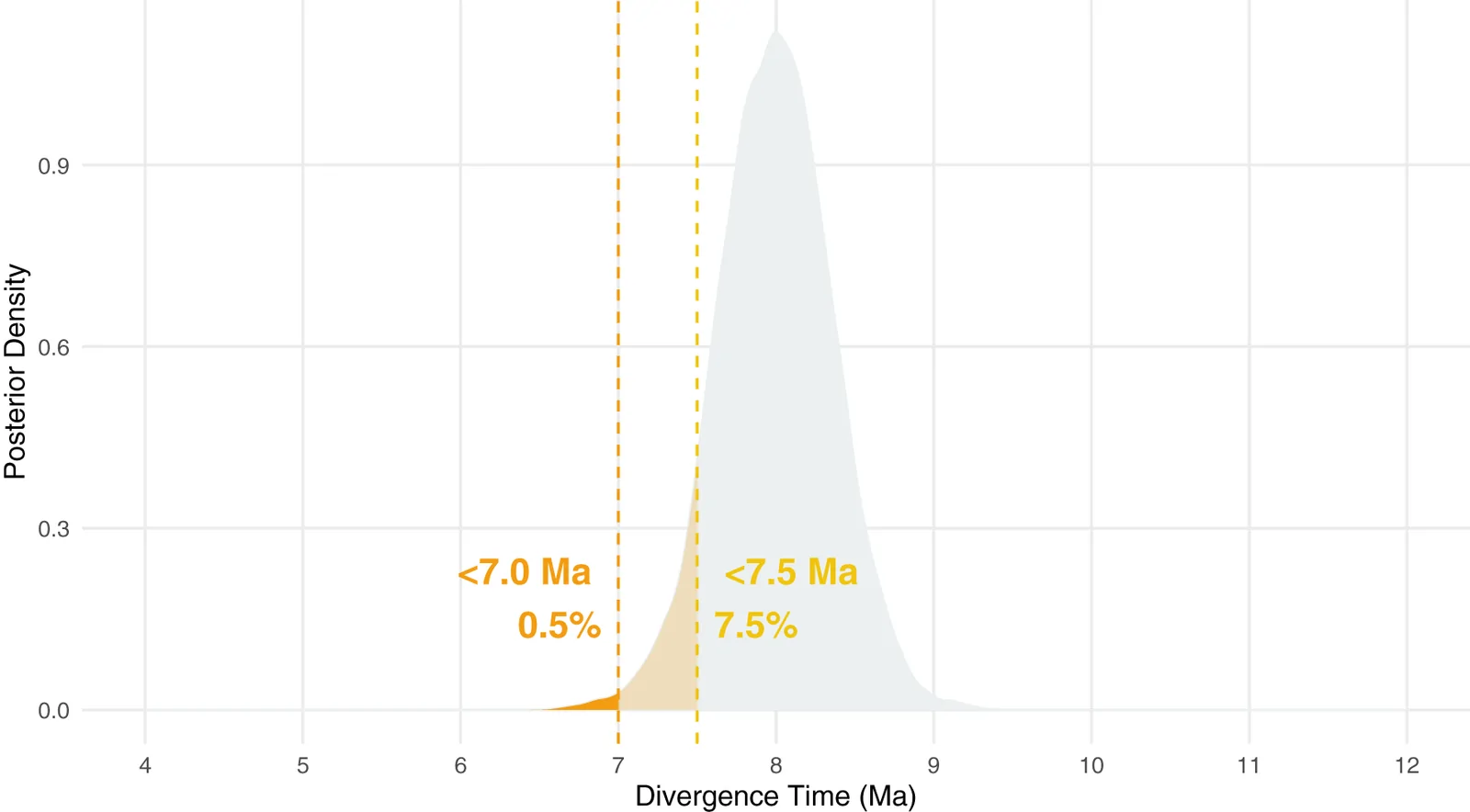
Posterior distribution of the meta-genomic model. Probability of divergence at specific time intervals

According to the phylogenomic meta-model there is only a 0.5% chance that the *Pan*/*Homo* split occured at 7.0 Ma or later, and a 7.5% chance at 7.5 Ma or later (Fig. 6). In this type of approach, the effect sizes and credible intervals displayed in the forest plot (Fig. 5) do not represent the observed effect sizes (i.e., reported in the original studies), but the posterior effect size of each study estimated by the Bayesian model. For example, Venn et al. (2014), estimated the *Pan*/*Homo* divergence at 12.71 (14.18–11.25) Ma, but this was readjusted to 11.91 (13.34–10.48) Ma through the Bayesian method.

## Discussion

Meta-analysis of 202 molecular estimates reveals a clear historical shift from the 6–4 Ma intervals popularized in early studies toward a late Miocene divergence. Integrating palaeontological thresholds demonstrates that as more fossil evidence is incorporated, estimates move deeper into the Miocene, reaching a mean of 9.88 Ma in the most restrictive scenario. Most significantly, our phylogenomic meta-model identifies a pooled divergence of 8.0 Ma (8.69–7.40 Ma), indicating a 99.5% posterior probability that the split predates 7.0 Ma. These results confirm that molecular timing is rapidly converging with the current fossil record, effectively reconciling decades of interdisciplinary discrepancy.

In 1982 there was a meeting organized at the Vatican that “led to a consensus between palaeontologists and molecularists: the divergence occurred at around 7 Ma” (Senut 2015, p. 2050) for the *Pan/Homo* pair. However, most molecular studies still indicate more recent divergence dates (median = 6.52 Ma; Table 1). In contrast, contemporary paleontological estimates, informed by improved late Miocene stratigraphic records, propose a significantly deeper split, with reported intervals including 12.8–10.1 Ma (Marshall 2019); ca. 12 Ma (Senut and Pickford 2004); 10–7 Ma (White et al. 2009); 9.5–7.5 Ma (Haile-Selassie et al. 2009); and 10–6.5 Ma (Benton and Donoghue 2007). Taken together these palaeontological estimates average 9.48 Ma. Considering this difference of roughly 2.96 million years between the average estimates of molecular biologists and palaeoanthropologists, it is evident that although a complete consensus remains elusive, there is a noticeable trend toward greater convergence between the two fields.

The idea that hominins must have diverged at around 6–4 Ma has prevailed for many decades, even though hundreds of molecular clock estimates have been published after Sarich and Wilson (1967) with increasingly sophisticated methods and higher resolution datasets. Despite computational and data improvements, molecular dating remains challenged by fundamental assumptions often violated in practice, including: guaranteed homology, straightforwardly measurable molecular change, accurate capture of rate variation, random sequence sampling, constant speciation-extinction rates, and the primacy of data over model priors or calibrations (Bromham 2019). The assumption of constant substitution rates in molecular clock models, dominant for over four decades, has also proven problematic. Rates vary with factors such as body size, population dynamics, generation time, overlapping generations, and other ecological and life-history traits, all of which can change substantially over millions of years (Steiper et al. 2004; Elango et al. 2006; Bromham 2011). These issues have been amplified by the genomic era, where exponential growth in data can reduce the signal-to-noise ratio in analyses. While genomics has greatly expanded sequence data availability, it must be matched by comparable advances in modelling assumptions and methods (Kuderna et al. 2023; Rivas-González et al. 2023; Shao et al. 2023).

Generation time is likely the primary determinant of substitution rates (Steiper and Seiffert 2012). Specifically, due to the longer generation times observed in hominoids compared to other primates (Steiper et al. 2004), this effect is exacerbated in human generation times which are even longer when contrasted with other apes, and this reflects on substitution rates (Steiper and Young 2006). This biological reality makes divergence dates prone to underestimation unless adjusted via relaxed molecular clock models and refined calibrations (Schrago and Voloch 2013; dos Reis et al. 2018). Variation in substitution rates was largely overlooked until the early 2000s, with the advent of genomics lending support to the ‘hominoid slowdown hypothesis’ first proposed by Goodman (1961). This shift highlighted the limitations of conventional methods, which assume uniform evolutionary rates across species and thus fail to grasp the intricacies of hominin evolutionary dynamics (Yi 2013).

Minimum age constraints should be based on the oldest fossil evidence of the extant clades. Maximum ages, in constrast, can be estimated by probabilistic approaches that integrate a suite of fossil occurrences, together with palaeobiogeographic and taphonomic data (Tavaré et al. 2002; Marshall 2019; Du et al. 2020). However, establishing a maximum bound is more challenging, as reliance on fossil-based phylogenies (in contrast to genetic-based), and uncertainty in taxonomic assignments both increase with deeper time (Marshall 1990).

Minimum calibration values should account for updated geological dating of fossil material. For example, many divergence studies using *S. tchadensis* have adopted minimum bounds of 6 or 6.5 Ma despite revised age estimates placing the material at 7.2–6.8 Ma (Lebatard et al. 2008) and, with improved methods, at 7.34–7.1 Ma (Lebatard et al. 2010). Accordingly, *Sahelanthropus’* associated fauna (Anthracotheriid Unit, Chad) shows similarities with the faunal record from the lower Nawata Formation from Kenya which may be as old as 7.4 Ma (McDougall and Feibel 2003; dos Reis and Yang 2019). Because calibration points can impose hard minimum bounds, using underestimated values can skew results toward younger divergence times (Steiper et al. 2004; Marshall 2008). At the same time, the incompleteness of the fossil record means that minimum bounds remain uncertain, and this uncertainty should be explicitly incorporated into divergence time models (Yang and Rannala 2006).

All results indicate there is a bias toward younger estimates, in some cases directly contradicting the palaeontological record. In our dataset, mean estimates for the *Pan*/*Homo* split show a complete absence within the 9 and 9.5 Ma interval, despite a normal distribution predicting roughly ten to eleven studies in this range. Interestingly, the Bayesian meta-analysis using the most restrictive threshold (7.2 Ma), resulted in 8.66 Ma, which aligns more closely with the regression-based molecular trend from the full dataset (8.4 Ma, when = 2023) than with any of the other regressions.

The meta-phylogenomic analysis is based on a more standardized statistical framework and yields an estimate of 8.0 Ma. However, because studies reporting lower divergence times tend to have smaller SE than studies with higher estimates, meta-analytic weighting (inversely proportional to standard error) inherently favours younger values. As a result, conventional meta-analytic approaches may be ill-suited to molecular clock divergence data, as they systematically overweight younger estimates. This bias affects not only the pooled estimate of the model but also the posterior distributions and effect size of each study. In this study, the dataset was analysed using multiple statistical approaches to make up for the current shortcomings of meta-analysis with this type of data.

As for the validity of the fossil thresholds selected to work as minimum bounds, they depend on the taxonomic and geological dating rigour associated with each taxon (Püschel et al. 2021). Apomorphies that would fossilize and be detectable require some time to evolve after the divergence happened (Marshall 2008, 2017), and these may not be readily sampled given the fragmentary nature of the fossil record (Tavaré et al. 2002; Wilkinson et al. 2011). Therefore, some authors argue that estimates between 8 and 7 Ma are too recent and unlikely to be compatible with *Sahelanthropus*, *Orrorin* or *Ardipithecus* being hominins (Senut and Pickford 2004; Marshall 2019; Bobe and Wood 2022). Yet these multiple lineages of fossil evidence, which some researchers suggest might even represent a single genus (White et al. 2009), did not have much effect on reconsidering the assumptions and establishment of min-max bounds, despite their profound implications for the outcomes of molecular models. This is clearly evidenced by the majority of estimations published after 2002 (58.78%) still indicating divergence estimates more recent than the dates associated to the fossil material that might represent the earliest Hominina or Panina (Macchiarelli et al. 2020; Daver et al. 2022; Meyer et al. 2023; Williams et al. 2026).

Historically, some studies have rejected the hominin status of key fossil taxa based on molecular models. Instead, we need to consider the high variance associated with reported molecular estimates, and use the palaeontological record and geological work done in the late Miocene of Africa as tools to understand which of these models are compatible with the fossil evidence (Moorjani et al. 2016; Barba-Montoya et al. 2017). In this context, the hypothesis of a Pliocene divergence (< 5.3 Ma), once prominent in early molecular studies, is increasingly untenable, as it conflicts with multiple late Miocene fossil taxa (e.g., *Sahelanthropus*, *Orrorin*, *Ardipithecus*) that show accumulated evidence of hominin affinities. Accordingly, the field has shifted away from a Pliocene versus Miocene dichotomy toward refining the timing of the divergence within the late Miocene. Consistent with this trend, regression analyses indicate that molecular estimates have progressively converged toward values more compatible with the palaeontological record, and vice versa.

Overall, all analyses indicated that the late Miocene is the most likely geological period for the hominin divergence from the other African apes. However, this represents a large sub-epoch spanning 6.3 million years, which is more time than has passed since the end of the Miocene up to the present. Our study indicates that there is a structure to the published molecular data that can be used to increase the accuracy and precision of our estimates of when the hominin lineage first emerged.

The Bayesian meta-model employing only genomic data suggested that the split must have happened quite early in the late Miocene, with a 99.5% posterior probability of predating 7 Ma. These findings have significant implications for our understanding of hominin divergence and call for the need to target more fossil deposits covering the reported intervals to test and validate these estimations. Notably, the near absence of fossil sites preserving 9 to 7 Ma deposits across Africa represents a critical research gap that warrants thorough investigation and attention.

## Conclusions

1. Despite historical discrepancies, there is a trend toward greater convergence between molecular biology and palaeoanthropology in estimating *Pan*-*Homo* divergence.
2. Considering the patchiness of the fossil record, establishing accurate minimum age constraints for calibration points is crucial for improving divergence estimates.
3. Molecular models should incorporate up-to-date palaeontological record and geological data to align with the full spectra of scientific evidence.
4. All analyses suggest a late Miocene divergence, most likely before 7.5 Ma, but we are yet to find sites with 9 to 8 Ma fossils in Africa.

## Supplementary Information

Below is the link to the electronic supplementary material.

Supplementary Material 1
