# Supplementary material for: Parting ways: Pan-Homo divergence revisited
Source: Primates. 2026 Jun 18;67(5):549–65. doi: 10.1007/s10329-026-01269-w (PMC13541867; doi:10.1007/s10329-026-01269-w)
Supplement: Supplementary file 1 [file 10329_2026_1269_MOESM1_ESM.docx]

## **Supplementary Information**

### **S1: Dataset distribution**

Is the database generally biased toward values that are too recent in geological time to be possible? We would expect that the density curves of estimations (Fig. S1) would approximate a normal distribution and that their left tail should start close to the levels of the fossil thresholds (4.4 to 7.2 Ma). Moreover, they should center on some larger value and then mirror to some extent this pattern at the right tail i.e., normality. Instead, we obtained displaced (starting at ~1.3 Ma) and right-skewed density curves, especially for the Min range values. Overall, these statistical artefacts are strong evidence of a bias towards underestimation of time passed since the *Pan/Homo* divergence. As expected, the Max estimations are platykurtic—revealing uncertainty—since deriving a maximum bound from the fossil record is more problematic as it must rely on negative evidence (i.e., the absence of a palaeo-taxon in a stratigraphic horizon or time interval) (Marshall 1990).

**Fig. S1.**

Density curves for the min-estimate-max triplets sampled from the literature. Dashed vertical bars represent median values for each density curve.

### **S2: Bayesian meta-analysis: further details**

A Bayesian meta-analysis can be seen as a special case of a Bayesian multilevel regression (Higgins et al. 2009; Bürkner 2018; McElreath 2020; Röver 2020). By definition, a meta-analysis is structured hierarchically, i.e., it has an inherent multilevel architecture (Cumming 2013). In our case, the first level includes the individual estimates of divergence dates for the *Pan*/*Homo* pair. In a meta-analysis, data on this level is generally described as the calculated effect sizes $y_{i}$ of each study $i$. Since some studies include multiple model outputs with different divergence estimates, we assume that estimates are nested within studies on the second level. Thus, the true effect sizes $\theta_{i}$ of different studies in the second level of our meta-analysis follow their own distribution with a pooled effect $\mu$ and a variance $\tau^{2}$ (between-study heterogeneity).

To formalize our Bayesian multilevel model, it follows that

$$y_{i}\sim Normal(\theta_{i},\sigma_{i}^{2})$$

$$\theta_{i}\sim Normal(\mu,\tau^{2})$$

(eq. 1)

where we assumed that our effect sizes $y_{i}$ were sampled from a population of $i$ studies. This population can be estimated as having a distribution with mean $\theta_{i}$ (i.e., the “true” effect of each divergence estimate), and a variance $\sigma^{2}$. The pooled effect size, that is, what we want to estimate in a meta-analysis, is defined as the mean $\mu$ of the distribution of true effect sizes. The study-specific true effects $\theta_{i}$ will deviate from the population-mean $\mu$, because the second-level distribution has a variance $\tau^{2}$.

The objective of a Bayesian meta-analysis is to estimate a single pooled true effect from observed effect sizes available in the literature. Thus, it shares the same objective of any conventional meta-analysis (Röver 2020). However, there are clear advantages of using Bayesian approaches. The main one is that domain knowledge—in this case, palaeoanthropological expertise, and specifically, the dates provided by the fossil record—can be easily incorporated into our models, by assigning prior distributions on the population-level parameters $\mu$ and $\tau^{2}$. Prior distributions are used to account for the uncertainty in the parameter values.

We rely on informative priors to incorporate domain-specific knowledge, instead of the more commonly used non-informative or uniform priors (i.e., all effect sizes are equally credible) (Williams et al. 2018; Röver et al. 2021). These priors are derived empirically from our filtered datasets (e.g., Filter B), allowing the model to be guided by established paleontological and genomic ranges while still accounting for uncertainty. Assuming a normal distribution, we define our population-level mean ($\mu$) prior using the calculated average $u_{prior}$and standard deviation $\sigma_{prior}^{2}$from the respective data subsets.

Regarding between-study heterogeneity $\tau$, we employ a Student-t distribution restricted to positive values, which provides a robust and weakly informative framework for modeling variance across highly variable studies (Williams et al. 2018; Wołodźko 2020). This approach offers more stable convergence for our specific hierarchical structure while still accommodating the wide spread of divergence estimates observed in the literature.

$$\mu\sim Normal\left( u_{prior}, \sigma_{prior}^{2} \right)$$

$$\tau\sim Student-T\left( 3, 0, 2.5 \right), \tau>0$$

(eq. 2)

In our case, we directly calculated the prior beliefs from our dataset after pruning it by applying the estimating averages and standard deviations of the sub-set under analysis.

**Fig. S2.**

Forest plot depicting the specific effect size and sampled posterior distribution of each study after applying the 4.4 Ma threshold.

**Table S1.**

Summary statistics for the Bayesian meta-analysis for the 4.4 Ma threshold.

| 4.4 Ma threshold | Divergence \| SE ~ 1 + (1 \| Study) | |
| --- | --- | --- |
| Predictors | Estimates | CI (95%) |
| Intercept | 6.90 | 6.58 – 7.23 |
| Std. Dev. | 1.52 | 1.30 – 1.77 |
| Random Effects | | |
| τ_00_ _Study_ | 1.36 | |
| ICC | 0.62 | |
| N _Study_ | 111 | |
| Observations | 159 | |
| Conditional R^2^ | 0.652 | |

**Fig. S3.**

Forest plot depicting the specific effect size and sampled posterior distribution of each study after applying the 6.2 Ma threshold.

**Table S2.**

Summary statistics for the Bayesian meta-analysis for the 6.2 Ma threshold.

| 6.2 Ma threshold | Divergence \| SE ~ 1 + (1 \| Study) | |
| --- | --- | --- |
| Predictors | Estimates | CI (95%) |
| Intercept | 7.68 | 7.35 – 8.03 |
| Std. Dev. | 1.28 | 1.04 – 1.58 |
| Random Effects | | |
| τ_00_ _Study_ | 1.69 | |
| ICC | 0.50 | |
| N _Study_ | 79 | |
| Observations | 108 | |
| Conditional R^2^ | 0.572 | |

**Fig. S4.**

Forest plot depicting the specific effect size and sampled posterior distribution of each study after applying the 7.2 Ma threshold.

**Table S3.**

Summary statistics for the Bayesian meta-analysis for the 7.2 Ma threshold.

| 7.2 Ma threshold | *Divergence \| SE ~ 1 + (1 \| Study)* | |
| --- | --- | --- |
| Predictors | Estimates | CI (95%) |
| Intercept | 8.64 | 8.17 – 9.12 |
| Std. Dev. | 1.32 | 0.98 – 1.75 |
| Random Effects | | |
| τ_00_ _Study_ | 1.89 | |
| ICC | 0.46 | |
| N _Study_ | 43 | |
| Observations | 62 | |
| Conditional R^2^ | 0.534 | |

### Data S1.

Maintained and Available on GitHub: <https://github.com/Delvis/Pan-Homo>

### **References**

Bürkner P-C (2018) Advanced Bayesian Multilevel Modeling with the R Package brms. R J 10:395. https://doi.org/10.32614/RJ-2018-017

Cumming G (2013) Understanding the new statistics: Effect sizes, confidence intervals, and meta-analysis. Routledge, London, UK

Higgins JPT, Thompson SG, Spiegelhalter DJ (2009) A re-evaluation of random-effects meta-analysis. J R Stat Soc Ser A Stat Soc 172:137–159. https://doi.org/10.1111/j.1467-985X.2008.00552.x

Marshall CR (1990) The fossil record and estimating divergence times between lineages: Maximum divergence times and the importance of reliable phylogenies. J Mol Evol 30:400–408. https://doi.org/10.1007/BF02101112

McElreath R (2020) Statistical Rethinking: A Bayesian course with examples in R and Stan. CRC press, Boca Raton, FL

Röver C (2020) Bayesian Random-Effects Meta-Analysis Using the **bayesmeta** *R* Package. J Stat Softw 93:1–51. https://doi.org/10.18637/jss.v093.i06

Röver C, Bender R, Dias S, et al (2021) On weakly informative prior distributions for the heterogeneity parameter in Bayesian random‐effects meta‐analysis. Res Synth Methods 12:448–474. https://doi.org/10.1002/jrsm.1475

Williams DR, Rast P, Bürkner P-C (2018) Bayesian Meta-Analysis with Weakly Informative Prior Distributions. PsyArXiv

Wołodźko T (2020) extraDistr: Additional univariate and multivariate distributions
